# Upregulated PKM2 Protects Dopaminergic Neurons From Oxidative Damage Through Nrf2 Transactivation in an MPTP‐Induced Mouse Model of Parkinson's Disease

**DOI:** 10.1002/cns.70968

**Published:** 2026-06-01

**Authors:** Meng Mei, Qian‐qian Miao, Meng‐ke Li, Hao‐ran Wang, Jian‐hua Ding, Gang Hu, Yao Wei

**Affiliations:** ^1^ Department of Pharmacy Wuhan Children's Hospital (Wuhan Maternal and Child Healthcare Hospital), Tongji Medical College, Huazhong University of Science and Technology Wuhan China; ^2^ School of Pharmacy Nanjing University of Chinese Medicine Nanjing China; ^3^ Jiangsu Key Laboratory of Neurodegeneration, Department of Pharmacology Nanjing Medical University Nanjing China

**Keywords:** dopaminergic neurons, glutathione peroxidase 4, oxidative stress, Parkinson's disease, peroxiredoxin 1, pyruvate kinase M2

## Abstract

**Background:**

The death of dopaminergic neurons in the substantia nigra pars compacta (SNc) is the core defining pathological change of Parkinson's disease (PD). We previously showed that reducing neuronal pyruvate kinase M2 (PKM2) aggravates oxidative damage and accelerates dopaminergic neuron loss, although the mechanism remained unclear.

**Methods:**

In the MPTP mouse model, we evaluated antioxidant responses and neuronal survival after selectively deleting PKM2 in dopaminergic neurons or overexpressing PKM2. In MPP^+^‐treated primary neurons, we examined how ROS influences hnRNP A1/A2 and alternative splicing toward PKM2, and we tested reversibility by knocking down hnRNP A1/A2 or scavenging ROS.

**Results:**

Neuronal PKM2 knockdown abolished MPTP‐induced activation of Nrf2 target genes, exacerbated lipid peroxidation and DNA damage, and accelerated loss of dopaminergic neurons, whereas PKM2 overexpression restored the antioxidant response and mitigated neurodegeneration. Mechanistically, ROS generated by MPP^+^ increased hnRNP A1/A2, promoting alternative splicing toward PKM2 and elevating its abundance; conversely, hnRNP A1/A2 knockdown or ROS scavenging reversed this splicing shift and attenuated the antioxidant response.

**Conclusion:**

These findings delineate a signaling pathway in which ROS elevate hnRNP A1/A2, favor PKM2 production, and activate Nrf2, thereby providing a mechanistic basis for the oxidative injury and progressive dopaminergic neuron degeneration observed with PKM2 loss. The PKM2–Nrf2 axis thus emerges as a candidate target for disease‐modifying therapy in PD.

## Graphical abstract

Oxidative stress induced by MPTP promotes hnRNP A1/A2–dependent alternative splicing toward PKM2 in dopaminergic neurons. Elevated PKM2 transactivates Nrf2, enhances antioxidant gene expression, and protects neurons from oxidative damage and degeneration. Disruption of the PKM2–Nrf2 axis exacerbates oxidative injury, identifying it as a potential disease‐modifying target in Parkinson's disease.

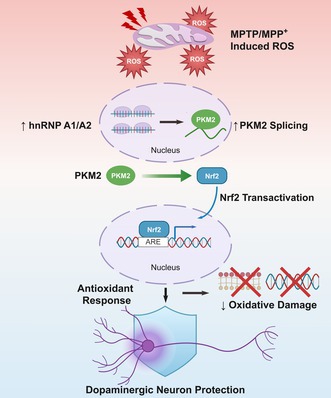

## 1 Introduction

Parkinson's disease (PD) ranks as the second most prevalent neurodegenerative disorder, with the fastest‐growing incidence rate [1]. Its defining pathological characteristics encompass the progressive degeneration of dopaminergic neurons in the substantia nigra pars compacta (SNc) and the widespread accumulation of Lewy bodies [2, 3]. Researchers have demonstrated that the etiology of PD is driven by a combination of genetic mutations and exposure to environmental toxins. Pathogenic mutations in genes such as SNCA [4, 5], LRRK2 [6], DJ‐1 [7, 8], Parkin [9], and PINK1 [10], as well as exposure to environmental toxins like 1‐methyl‐4‐phenyl‐1,2,3,6‐tetrahydrodropyridine (MPTP), rotenone, paraquat, dieldrin, chlorpyrifos, maneb, and permethrin, contribute to oxidative stress by impairing mitochondrial function and subsequently generating reactive oxygen species (ROS) [11, 12, 13]. Dopaminergic neurons are intrinsically vulnerable to oxidative damage due to their long, unmyelinated axons and numerous synapses [14, 15]. ROS oxidizes dopamine, initiating a time‐dependent pathological cascade that ultimately leads to the loss of dopaminergic neurons and the clinical symptoms associated with PD [16].

MPTP is a commonly employed reagent for establishing a robust mouse model of PD. It can cross the blood–brain barrier and is primarily metabolized by monoamine oxidase B within astrocytes. This metabolic process converts MPTP into 1‐methyl‐4‐phenyl‐2,3‐dihydropyridinium (MPDP^+^). Subsequently, MPDP^+^ undergoes auto‐oxidation to form the toxic metabolite, 1‐methyl‐4‐phenylpyridinium (MPP^+^). Notably, MPP^+^ is preferentially transported into dopaminergic neuron terminals, relying on the dopamine transporter for cellular entry. Once inside the neurons, intracellular MPP^+^ exerts its detrimental effects by inhibiting mitochondrial complex I of the respiratory chain. This inhibition leads to the production of substantial amounts of ROS, ultimately resulting in impairment of dopaminergic neurons [17, 18].

Nuclear factor erythroid 2‐related factor 2 (Nrf2), distinguished by its basic leucine zipper (bZIP) structural motif, functions as a central coordinator in cellular responses to oxidative stress. Following dissociation from its inhibitory binding partner Keap1, this transcription factor rapidly relocates to the nuclear compartment where it initiates the genetic activation of multiple pathways. The targeted gene network encompasses enzymatic components essential for glutathione biosynthesis, lipid peroxide neutralization, and general reactive oxygen species clearance, such as glutathione peroxidase 4 (GPX4) and peroxiredoxin 1 (PRDX1) [19, 20]. Recent research supports the notion that disturbances in Nrf2 expression and activity are implicated in the pathogenesis of a broad spectrum of diseases, spanning cancer, autoimmune diseases, metabolic disorders, respiratory illnesses, cardiovascular conditions, and neurodegenerative diseases like PD [21, 22]. Evidence from human brain samples indicates a heightened nuclear accumulation of Nrf2 within vulnerable dopaminergic neurons of patients with PD, suggesting a compensatory mechanism that enhances antioxidant capacity [23, 24]. Moreover, animal studies have shown that compounds like ellagic acid, resveratrol, and dapagliflozin protect rodents with PD from oxidative stress damage by restoring or activating Nrf2 [25, 26, 27]. Therefore, antioxidant therapy targeting Nrf2 activation is considered a promising treatment strategy for PD [21, 28].

Pyruvate kinase M2 (PKM2), beyond its established role as the rate‐limiting glycolytic enzyme responsible for the final ATP‐generating step [29], possesses non‐metabolic functions. Notably, it can also operate as a protein kinase and serve as a coactivator for various transcription factors [30, 31, 32]. Mounting evidence suggests that PKM2 plays a significant role in neurological disorders. It participates in the pathophysiology of conditions such as Alzheimer's disease, Huntington's disease, amyotrophic lateral sclerosis, and traumatic brain injury by modulating cellular metabolism, oxidative stress, neuroinflammation, and mitophagy [33, 34, 35, 36, 37]. Our previous work showed that reducing neuronal PKM2 aggravates oxidative injury and accelerates progressive degeneration of dopaminergic neurons [38], yet the mechanistic basis for this vulnerability remained unclear. Here we test the mechanistic hypothesis that neuronal PKM2, analogous to its role in astrocytes [39], activates Nrf2 and its antioxidant program to protect neurons. Our findings delineate a pathway linking oxidative stress to PKM2 abundance and Nrf2‐driven antioxidant defense, thereby explaining how PKM2 loss precipitates progressive dopaminergic neurodegeneration and nominating the PKM2–Nrf2 axis as a candidate target for disease‐modifying strategy in PD.

## 2 Materials and Methods

### 2.1 Animals

Male C57BL/6J mice were purchased from Hubei biont bio‐technology Co. Ltd. PKM2^flox/flox^ mice (JAX Stock: 024048) were a gift from Prof. Ke Zen of China Pharmaceutical University. Male mice are preferentially selected for PD modeling, as females show estrous cycle‐driven hormonal fluctuations and estrogen‐dependent dopaminergic neuroprotection resulting in blunted PD phenotypes and increased data variability. Males, by comparison, show greater sensitivity to PD‐causing neurotoxins, leading to marked dopamine loss and nigrostriatal injury that mirrors the clinical sex bias of higher PD prevalence in males. Their stable androgen levels guarantee experimental reproducibility, and alignment with published research promotes cross‐study comparability [40, 41, 42, 43, 44]. All mice were kept under specific pathogen‐free facility with a controlled ambient temperature at 22°C–24°C, a 12‐h light–dark photoperiod, and free access to standard rodent diet and water. Thirty PKM2^flox/flox^ mice received bilateral SNc microinjections with AAV‐TH‐CTL in the left hemisphere and AAV‐TH‐Cre (Obio Technology, Shanghai, China) in the right, and were divided into saline and MPTP‐treated subgroups (*n* = 15 each). Thirty wild type mice received bilateral SNc microinjections with AAV‐TH‐CTL in the left hemisphere and AAV‐TH‐PKM2 (Hanbio Biotechnology, Shanghai, China) in the right, and were similarly split into saline and MPTP‐treated subgroups (*n* = 15 each).

### 2.2 Adeno‐Associated Virus (AAV) Stereotaxic Microinjection

For AAV stereotaxic microinjection, the mice were anesthetized with pentobarbital sodium (60 mg/kg, i.p.) [45, 46] and fixed on a stereotaxic apparatus. We then shaved the mice head, sterilized the scalp, and drilled the skull. AAV‐TH‐Cre (Obio Technology, Shanghai, China) was microinjected into the SNc region of PKM2^flox/flox^ mice at the following coordinates: AP, −3.0 mm; ML, ±1.3 mm; DV, −4.2 mm (relative to the bregma). AAV‐TH‐PKM2 (Hanbio Biotechnology, Shanghai, China) was similarly microinjected into the SNc region of wild type mice. AAV‐TH‐CTL was microinjected into the SNc region for use as a control. All AAVs were microinjected at a rate of 0.25 μL/min, followed by a 5‐min needle retention prior to slow withdrawal [47, 48]. After suturing and disinfecting the scalp, the mice were transferred to a thermostatically controlled warming pad until they regained consciousness. Three weeks later, the mice were used for subsequent experiments.

### 2.3 MPTP‐Induced Mouse Subacute PD Model

The mice were subcutaneously injected with saline‐dissolved MPTP (20 mg/kg, HY‐15608, MedChemExpress, New Jersey, USA) once daily for five consecutive days [39, 49, 50]. Three days after the last injection, the mice were anesthetized, and the chest was opened to expose the heart. Sequentially, the heart was perfused with normal saline, and brain tissues were harvested after the perfusion.

### 2.4 Primary Cell Culture and Treatment

Primary neuron culture was performed as previously described [38]. Primary neurons were treated with a concentration gradient of MPP^+^ (0, 0.1, 1, 10 μM; D048, Sigma, Germany) for 24 h. To achieve overexpression or knockdown, primary neurons were infected with an adenovirus carrying mouse‐PKM2 (Hanbio Biotechnology, Shanghai, China) or lentivirus carrying mouse‐sh‐heterogeneous nuclear ribonucleoprotein A1 (hnRNP A1) and mouse‐sh‐heterogeneous nuclear ribonucleoprotein A2 (hnRNP A2) at varying multiplicity of infection (MOI) for 48 h. The shRNA sequences were as follows: mouse‐sh‐hnRNP A1 5′‐AACCTTGATGTCGTTAAACTG‐3′, mouse‐sh‐hnRNP A2 5′‐GTCACAATGCAGAAGTTAGAA‐3′. After virus infection, primary neurons were treated with MPP^+^ (10 μM) for 24 h. For the N‐Acetylcysteine (NAC, HY‐B0215, MedChemExpress, New Jersey, USA) groups, primary neurons were pretreated with NAC (100 μM) for 30 min before with MPP^+^ stimulation.

Primary astrocyte and microglia culture was performed as previously described [39, 48]. The NC and mouse‐sh‐PKM2 were transfected to astrocyte or microglia using Lipofectamine3000 (L3000015, Invitrogen, California, USA) according to the manufacturer's protocol. The mouse‐sh‐PKM2 sequence was 5′‐GGCAGAGGCUGCCAUCUACTT‐3′. Human‐si‐PKM2 was transfected into SH‐SY5Y cells, and the siRNAs sequence were as follows:

Human‐siPKM2‐1 sense: 5′‐GCUGUGCUAGACACUAAA‐3′, antisense: 5′‐UUUAGUGUCUAGAGCCACAGC‐3′; Human‐siPKM2‐2 sense: 5′‐CGGGUGAACUUUGCCAUGAAU‐3′, antisense: 5′‐AUUCAUGGCAAAGUUCACCCG‐3′; Human‐siPKM2‐3 sense: 5′‐GUUCGGAGGUUUGAUGAAAUC‐3′, antisense: 5′‐GAUUUCAUCAAACCUCCGAAC‐3′.

### 2.5 Tyramide Signal Amplification (TSA) Multispectral Staining

TSA multispectral staining was performed as previously described [38]. The antibodies used were as follows: PRDX1 (1:200, ab109498, Abcam, Cambridge, UK), GPX4 (1:100, ab125066, Abcam, Cambridge, UK), PKM (1:100, ab137791, Abcam, Cambridge, UK), hnRNP A1 (1:200, ab5832, Abcam, Cambridge, UK), hnRNP A2 (1:1000, ab259894, Abcam, Cambridge, UK), PKM1 (1:200, 7067, Cell Signaling Technology, Massachusetts, USA), PKM2 (1:200, 4053, Cell Signaling Technology, Massachusetts, USA), 4‐hydroxynonenal (4‐HNE, 1:25, ab48506, Abcam, Cambridge, USA), γ‐H2AX (1:200, ab2893, Abcam, Cambridge, USA), Cre (1:200, 15,036, Cell Signaling Technology, Massachusetts, USA), tyrosine hydroxylase (TH, 1:200 GB11181, Servicebio, Wuhan, China), HRP‐conjugated anti‐mouse IgG antibody (1:1000, SA00001‐1, Proteintech, Chicago, USA), and HRP‐conjugated anti‐rabbit IgG antibody (1:1000, SA00001‐2, Proteintech, Chicago, USA).

### 2.6 Immunohistochemistry Staining

The brain slices were immersed in 3% H_2_O_2_ at RT for 15 min to quench endogenous peroxidase activity, blocked and permeabilized with 5% bovine serum albumin (BSA)‐0.3% Triton X‐100 in PBS at RT for 1.5 h, and incubated with TH primary antibody (1:200, 58,844, Cell Signaling Technology, Massachusetts, USA) at 4°C overnight. After being rinsed with PBS, the brain slices were incubated with HRP‐conjugated anti‐rabbit IgG antibody (1:1000, SA00001‐2, Proteintech, Chicago, USA) at RT for 1.5 h, washed with PBS, and visualized with 3,3′‐diaminobenzidine (DAB‐0031, MXB Biotechnologies, Fuzhou, China). The brain slices were dehydrated through a graded ethanol series, cleaned with xylene, and finally mounted with neutral gum. TH‐positive cells were counted using the Stereo Investigator software (Olympus BX51, Tokyo, Japan).

### 2.7 Immunofluorescence Staining

Primary neurons were fixed with 4% paraformaldehyde at RT for 30 min, blocked and permeabilized with 5% BSA‐0.3% Triton X‐100 in PBS at RT for 1.5 h, followed by incubation with MAP2 primary antibody (1:200, 17,490–1‐AP, Proteintech, Chicago, USA) at 4°C overnight. After rinsing with PBS, primary neurons were incubated with anti‐rabbit IgG (H + L), F(ab’)2 Fragment (Alexa Fluor 488 Conjugate) (1:1000, 4412, Cell Signaling Technology, Massachusetts, USA) at RT for 1.5 h in the dark and imaged using confocal microscopy (STELLARIS 5, Leica, Wetzlar, Germany). For neurite length quantification, 5 random fields were selected per mouse, with 20 morphologically intact neurons analyzed per field. The neurite of each selected neuron was manually traced from the cell body to its distal tip using the Neuron J plugin in Image J software to determine individual neurite lengths.

### 2.8 Western Blot and co‐Immunoprecipitation (Co‐IP)

Lysis of cells was performed by incubation in RIPA lysis buffer supplemented with protease and phosphatase inhibitors on ice for 30 min. Nuclear protein was extracted from the primary neurons using a nuclear and cytoplasmic protein extraction kit (P0028, Beyotime Biotechnology, Shanghai, China) according to the manufacturer's protocol. The sample lysates were centrifuged at 16,000 *g* for 10 min at 4°C and the supernatants were aspirated. The protein concentration of the supernatants was quantified using the BCA protein assay kit (P0012S, Beyotime Biotechnology, Shanghai, China) according to the manufacturer's protocol. The protein concentrations in the samples were normalized by diluting them with RIPA lysis buffer as required. The supernatants were added with the protein loading buffer and denatured. Protein samples were separated by SDS‐PAGE and subsequently transferred onto PVDF membranes. Following blocking with 5% skimmed milk for 1 h at room temperature, the membranes were incubated with primary antibodies overnight at 4°C. After extensive washing with TBST, the membranes were probed with HRP‐conjugated secondary antibodies for 1.5 h at room temperature. Protein bands were finally visualized using an enhanced chemiluminescence substrate (BL523A, Biosharp Life Science, Hefei, China) and imaged using the gel imaging system (ChemiDoc XRS+, BioRad, California, USA). Antibodies used in this experiment were as follows: hnRNP A1 (1:1000, 8443, Cell Signaling Technology, Massachusetts, USA), hnRNP A2 (1:1000, ab259894, Abcam, Cambridge, UK), PKM1 (1:1000, 15,821–1‐AP, Proteintech, Chicago, USA), PKM2 (1:1000, 4053, Cell Signaling Technology, Massachusetts, USA), PKM (1:1000, 3190, Cell Signaling Technology, Massachusetts, USA), PRDX1 (1:1000, 8499, Cell Signaling Technology, Massachusetts, USA), GPX4 (1:1000, 52,455, Cell Signaling Technology, Massachusetts, USA), 4‐HNE (1:500, MA5‐27570, Invitrogen, Massachusetts, USA), γ‐H2AX (1:1000, 9718, Cell Signaling Technology, Massachusetts, USA), Nrf2 (1:500, 12,721, Cell Signaling Technology, Massachusetts, USA), Superoxide dismutase 2 (SOD2, 1:5000, 24,127–1‐AP, Proteintech, Chicago, USA), TP53‐induced glycolysis and apoptosis regulator (TIGAR, 1:1000, 22,136‐1‐AP, Proteintech, Chicago, USA), GAPDH (1:3000, 60,004–1‐Ig, Proteintech, Chicago, USA), Lamin B1 (1:1000, 66,095–1‐Ig, Proteintech, Chicago, USA), β‐actin (1:2000, EM21002, HUABIO, Hangzhou, China), HRP‐conjugated anti‐mouse IgG antibody (1:2000, SA00001‐1, Proteintech, Chicago, USA), and HRP‐conjugated anti‐rabbit IgG antibody (1:2000, SA00001‐2, Proteintech, Chicago, USA).

For Co‐IP, primary neurons were lysed on ice with IP lysis buffer supplemented with protease and phosphatase inhibitors. An appropriate amount of total protein from the lysate was incubated with anti‐Nrf2 antibody, anti‐PKM2, or mouse IgG at 4°C overnight with rotation. Protein A/G agarose was then added for further incubation at 4°C for 4 h with rotation. The mixture was centrifuged, the supernatant was discarded, and the pellet was washed with pre‐cooled PBS. Finally, 2× loading buffer was added, and samples were boiled at 95°C for 5 min before loading. Subsequent steps followed the western blot protocol.

### 2.9 Blue Native Page (BNP)

Dimeric and tetrameric PKM2 were detected by BNP as previously described [39]. Total proteins were extracted with BNP lysis buffer (50 mM BisTris‐HCl, 0.5 M 6‐aminocaproic acid, 10% glycerol, 1% digitonin, pH 7.0) supplemented with protease and phosphatase inhibitor cocktails, followed by lysis on ice for 30 min. Lysates were centrifuged at 16,000 g and 4°C for 15 min, and supernatants were collected. Protein concentrations were quantified via BCA assay, normalized with BNP lysis buffer, and mixed with 2× BNP loading buffer. Samples were loaded onto BNP gels and electrophoresed at 4°C and 100 V using anode and cathode buffers. Proteins were transferred to PVDF membranes, decolorized with methanol, and subsequent steps were performed as described for western blot.

### 2.10 qRT‐PCR

Total RNA of the primary cells was extracted using the FastPure Cell/Tissue Total RNA Isolation Kit V2 (RC112, Vazyme, Nanjing, China) and reverse‐transcribed using HiScript III RT SuperMix for qPCR (+gDNA wiper) (R323‐01, Vazyme, Nanjing, China) according to the manufacturer's protocol. Real‐time quantitative PCR was conducted in a 10 μL mix system containing SYBR (Q711‐02, Vazyme, Nanjing, China), primer pairs, and cDNA, and analyzed using the ABI Q3 system (Thermo Fisher Scientific, Massachusetts, USA). PCR amplification was performed under the following conditions: stage 1: 95°C for 30 s, Reps 1; stage 2: 95°C for 10 s, 60°C 30 s, Reps 40; stage 3: 95°C for 15 s, 60°C 1 min, 95°C 15 s, Reps 1. Relative quantification of mRNA expression was calculated via the 2^−ΔΔ*Ct*^ formula, with β‐actin serving as the endogenous reference gene for normalization. The primers were self‐designed using NCBI Primer‐BLAST and commercially synthesized by TsingkeBiotechnology Co. Ltd. Primer sequences are as follows:

mouse‐PKM: forward 5′‐GTGATGTGGCCAATGCAGTC‐3′, reverse 5′‐CGGCGGAGTTCCTCGAATAG‐3′; mouse‐PKM1: forward 5′‐CACCGTCTGCTGTTTGAAGA‐3′, reverse 5′‐TCAAAGCTGCTGCTAAACACTT‐3′; mouse‐PKM2: forward 5′‐AGGCTGCCATCTACCACTTG‐3′, reverse 5′‐CACTGCAGCACTTGAAGGAG‐3′; mouse‐GPX4: forward 5′‐GCCAAAGTCCTAGGAAACGC‐3′, reverse 5′‐CCGGGTTGAAAGGTTCAGGA‐3′; mouse‐PRDX1: forward 5′‐GGTATCTCTTTCAGGGGCCTTT‐3′, reverse 5′‐TCTTCTGGCTGCTCAATGCT‐3′; mouse‐GPX2: forward 5′‐CACTGTTTCCCCTGAGCAGT‐3′, reverse 5′‐CCCAAGCAAACTCCCCAAGA‐3′;mouse‐GSR1: forward 5′‐GCCGCCTGAACACCATCTAT‐3′, reverse 5′‐CGAGGACCATCTGCGAATGT‐3′; mouse‐GSTA1: forward 5′‐CCGTTACTTGCCTGCCTTTG‐3′, reverse 5′‐ATTGGGGAGGCTGCTGATTC‐3′; mouse‐HO1: forward 5′‐GCTGCTCGCTCACGGT‐3′, reverse 5′‐GATTCAGGCTCCGGGCTATG‐3′; mouse‐GCLC: forward 5′‐GCAGCTTTGGGTCGCAAGTAG‐3′, reverse 5′‐TGGGTCTCTTCCCAGCTCAGT‐3′; mouse‐GCLM: forward 5′‐TTGCTGCTCAGTTGGACTCA‐3′, reverse 5′‐CGCCTTTCCTGGCTTTACTC‐3′; mouse‐G6PDH: forward 5′‐CCACTCCAGAAGAAAGACCTAAG‐3′, reverse 5′‐TGGCTGTTGAGGTGCTTATAG‐3′; mouse‐NQO1: forward 5′‐AGGGCAGAAGGGAATTGCTC‐3′, reverse 5′‐AAAGAGCTGGAGAGCCAACC‐3′; and mouse‐β‐actin: forward 5′‐GGACTGTTACTGAGCTGCGTT‐3′, reverse 5′‐CGCCTTCACCGTTCCAGTT‐3′.

### 2.11 Pyruvate Kinase mRNA Splicing Assay

Total RNA was extracted and reverse‐transcribed into cDNA as described for qRT‐PCR. Briefly, 2 μg of total RNA was extracted from the primary neurons and reverse transcribed into cDNA. The cDNA was amplified using the 2 × Taq Master Mix (P111, Vazyme, Nanjing, China). The amplification products were aliquoted into four equal volumes and then cleaved with PstI (NEB‐R3140S, New England Biolabs, Massachusetts, USA), NcoI (NEB‐R3193S, New England Biolabs, Massachusetts, USA), both PstI and NcoI, or neither enzyme, at 37°C for 30 min. The enzyme‐digested products were loaded and separated using 1.5% agarose gel electrophoresis and visualized using the gel imaging system (ChemiDoc XRS+, BioRad, California, USA). The primer sequences of mouse‐PKM were as follows: forward 5′‐ATGCTGGAGAGCATGATCAAGAAGCCACGC‐3′, reverse 5′‐CAACATCCATGGCCAAGTT‐3′ [51].

### 2.12 Chromatin Immunoprecipitation (ChIP)

ChIP assays were performed using the Chromatin Immunoprecipitation Assay Kit (17–295, Millipore, Massachusetts, USA). Primary neurons were crosslinked with 1% formaldehyde at 37°C for 20 min, and the reaction was quenched with 0.125 M glycine. Cells were harvested, lysed, and sonicated on ice to shear chromatin into fragments. Lysates were pre‐cleared with Salmon Sperm DNA/Protein A agarose, then incubated overnight at 4°C with anti‐Nrf2 antibody. Immune complexes were captured, washed sequentially, eluted, and incubated at 65°C for 4 h to reverse crosslinks. Following protein K treatment at 45°C for 1 h, DNA fragments were purified and analyzed by qRT‐PCR. Primer sequences are as follows:

mouse‐GPX4: forward 5′‐CAAATGGGAATACAGTGGCTC‐3′, reverse 5′‐CTGAGGCTGGAGTTAGACAC‐3′; mouse‐GPX4‐NC: forward 5′‐ATCCATCTTGTAGTTGCCTGG‐3′, reverse 5′‐AGATCTGACACCCTCTCCTG‐3′; mouse‐PRDX1: forward 5′‐AGCTGCCTATTAGAAGCATTC‐3′, reverse 5′‐GTACTTCTACTTGCCCAACC‐3′; mouse‐PRDX1‐NC: forward 5′‐GAAGCCCCATAGGAGACACA‐3′, reverse 5′‐CCACAAGTTCCACCATGTGTT‐3′.

### 2.13 Statistical Analysis

Data analysis was conducted with Prism 9.0 software (GraphPad, California, USA). All data were first assessed for normality using the Shapiro–Wilk test. For normally distributed data, parametric tests were applied as follows: comparisons between two groups were made using an unpaired Student's *t*‐test; comparisons among multiple groups for one single factor were conducted using one‐way analysis of variance followed by Tukey's post hoc test; and comparisons involving multiple factors were performed using two‐way ANOVA followed by Tukey's post hoc test. For data violating the normality assumption, appropriate non‐parametric tests were applied. The statistical analysis methods were selected according to the test requirements. Data are presented as mean ± SEM. A *p* < 0.05 was deemed statistically significant.

## 3 Results

### 3.1 Dopaminergic Neuron‐Specific PKM2 Deficiency Impairs Nrf2 Activation in an MPTP‐Induced PD Model

**FIGURE 1:**
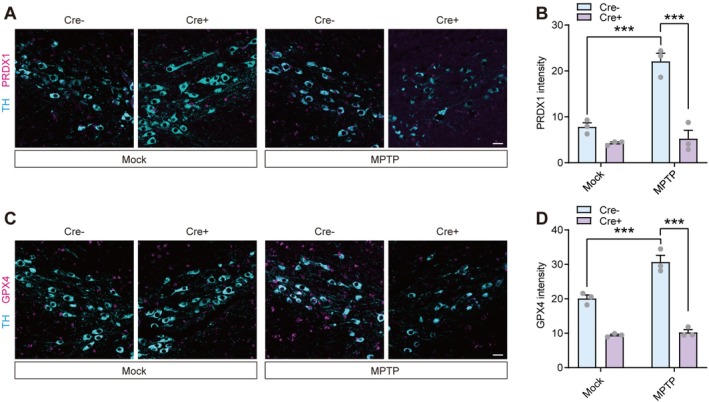
Dopaminergic neuron‐specific PKM2 deficiency impairs Nrf2 activation in an MPTP‐induced PD model. (A, B) Representative images and quantification of TSA multispectral staining of PRDX1 in TH‐positive neurons from saline or MPTP‐treated PKM2^flox/flox^ mice that were stereotaxically injected with AAV‐TH‐Cre or AAV‐TH‐CTL in the SNc. *n* = 3 mice per group. (C, D) Representative images and quantification of TSA multispectral staining of GPX4 in TH‐positive neurons from saline or MPTP‐treated PKM2^flox/flox^ mice that were stereotaxically injected with AAV‐TH‐Cre or AAV‐TH‐CTL in the SNc. *n* = 3 mice per group. Scale bar = 50 μm. ***p < 0.001. Two‐way analysis of variance followed by Tukey's post hoc test (B and D). Data are presented as mean ± SEM. GPX4, Glutathione peroxidase 4; MPTP, 1‐m ethyl‐4‐phenyl‐1,2,3,6‐tetrahydrodropyridine; PRDX1, Peroxiredoxin 1; TH, Tyrosine hydroxylase.

Given that reducing neuronal PKM2 aggravates oxidative injury and accelerates progressive loss of dopaminergic neurons [38], we first asked whether PKM2 is required to activate the Nrf2 antioxidant program in these neurons in vivo. To explore the mechanism underlying this regulation, we next examined whether PKM2 associates with Nrf2 in neurons. Co‐immunoprecipitation assays in primary neurons revealed a specific endogenous interaction between PKM2 and Nrf2 (Figure S1A). We therefore proceeded to ablate PKM2 in dopaminergic neurons in vivo to determine its requirement for Nrf2‐dependent antioxidant activation. We employed PKM2^flox/flox^ mice and performed unilateral microinjections of AAV‐TH‐Cre into the SNc. Simultaneously, the contralateral SNc of the same PKM2^flox/flox^ mice was microinjected with the control AAV‐TH‐CTL. Subsequently, we assessed the specificity of Cre recombinase and the efficiency of PKM2 knockdown. TSA multispectral staining results demonstrated that Cre recombinase was specifically and highly expressed in TH‐positive neurons (Figure S1C,D), resulting in a significant reduction in PKM2 expression within TH‐positive neurons (Figure S1E,F). We then induced a PD model in mice using MPTP to assess the expression of Nrf2 downstream targets, PRDX1 and GPX4 (Figure S1B). We observed that MPTP stimulation led to an increase in the expression of these two proteins in TH‐positive neurons in the SNc. However, PKM2 deficiency eliminated this increase (Figure 1A–D), indicating the essential role of PKM2 in Nrf2 activation within dopaminergic neurons in the MPTP‐induced PD model.

### 3.2 Dopaminergic Neuron‐Specific PKM2 Deficiency Exacerbates Neuronal Oxidative Stress and Loss in an MPTP‐Induced PD Model

**FIGURE 2:**
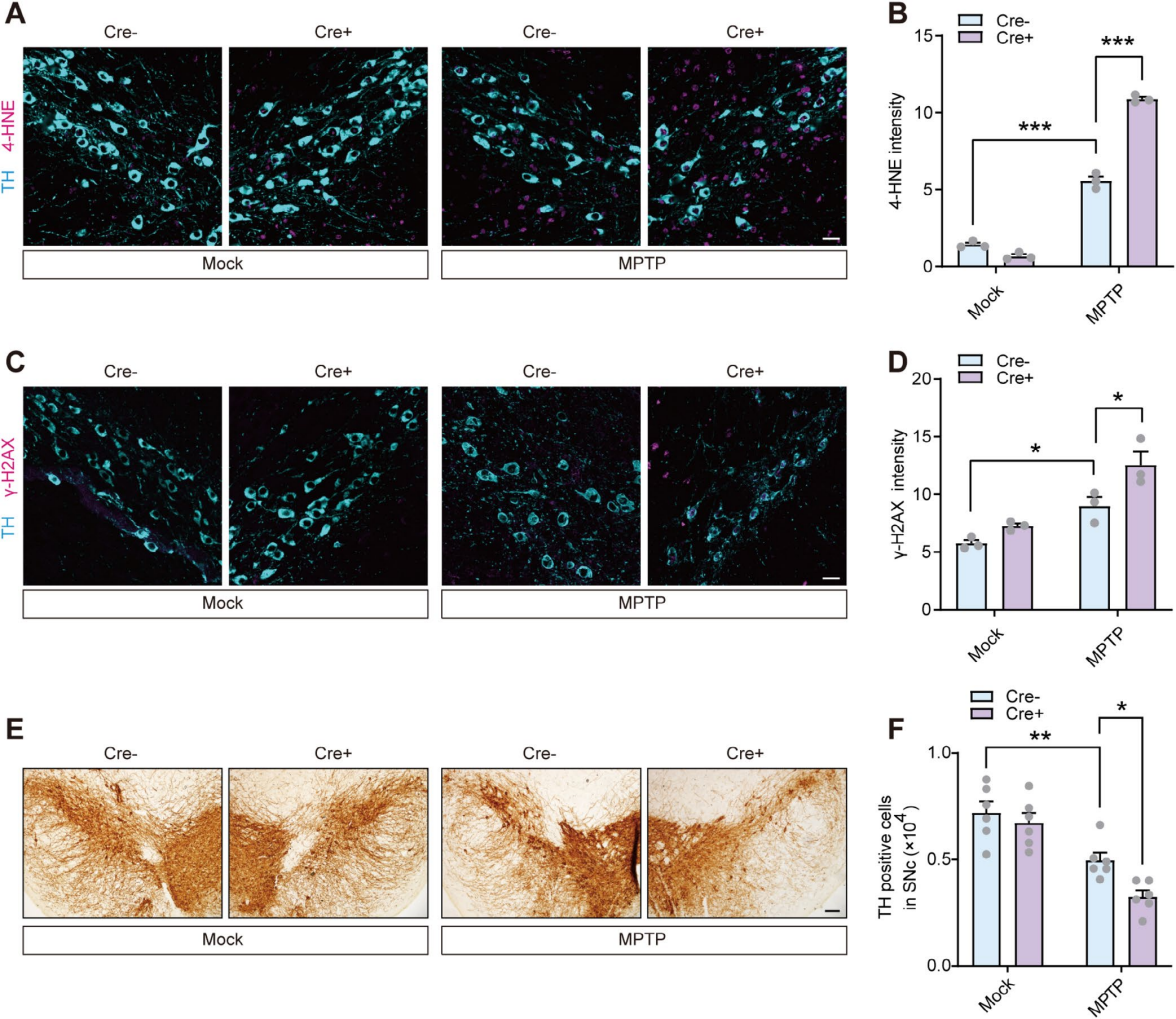
Dopaminergic neuron‐specific PKM2 deficiency exacerbates neuronal oxidative stress and loss in an MPTP‐induced PD model. (A, B) Representative images and quantification of TSA multispectral staining of 4‐HNE in TH‐positive neurons from saline or MPTP‐treated PKM2^flox/flox^ mice that were stereotaxically injected with AAV‐TH‐Cre or AAV‐TH‐CTL in the SNc. *n* = 3 mice per group. Scale bar = 50 μm. (C, D) Representative images and quantification of TSA multispectral staining of γ‐H2AX in TH‐positive neurons from saline or MPTP‐treated PKM2^flox/flox^ mice that were stereotaxically injected with AAV‐TH‐Cre or AAV‐TH‐CTL in the SNc. *n* = 3 mice per group. Scale bar = 50 μm. (E, F) Representative images and quantification of immunohistochemistry staining of TH‐positive neurons from saline or MPTP‐treated PKM2^flox/flox^ mice that were stereotaxically injected with AAV‐TH‐Cre or AAV‐TH‐CTL in the SNc. *n* = 6 mice per group. Scale bar = 100 μm. *p < 0.05, **p < 0.01, ***p < 0.001. Two‐way analysis of variance followed by Tukey's post hoc test (B, D, and F). Data are presented as mean ± SEM. 4‐HNE, 4‐hydroxynonenal; MPTP, 1‐methyl‐4‐phenyl‐1,2,3,6‐tetrahydrodropyridine; SNc, Substantia nigra pars compacta; TH, Tyrosine hydroxylase.

Nrf2 maintains cellular redox homeostasis by transcriptionally upregulating a network of antioxidant and cytoprotective genes. Hence, we investigated the extent of oxidative damage and subsequent dopaminergic neuron loss in response to PKM2 deficiency. Immunostaining results revealed that PKM2 deficiency heightened the accumulation of 4‐HNE produced by lipid peroxidation and γ‐H2AX, a biomarker of DNA double‐strand breaks, in TH‐positive neurons within the MPTP‐induced mouse model (Figure 2A–D). Consequently, this exacerbated the loss of dopaminergic neurons induced by MPTP (Figure 2E,F). These findings underscore that the inhibition of PKM2 intensifies neuronal oxidative damage and neuronal loss in the MPTP‐induced PD model. Given that PKM2 has also been implicated in the regulation of other redox‐related signaling pathways, we further examined whether PKM2 modulates NF‐κB‐ or p53‐associated antioxidant responses [52, 53, 54]. In MPP^+^‐challenged SH‐SY5Y cells with PKM2 knockdown, we measured the expression of SOD2, a representative NF‐κB downstream antioxidant gene [55, 56], and TIGAR, a well‐established p53 target involved in redox regulation [57]. PKM2 knockdown did not significantly alter SOD2 expression, whereas it reduced TIGAR expression upon MPP^+^ treatment (Figure S2). These results suggest that, in addition to Nrf2‐dependent antioxidant programs, PKM2 may also engage the p53–TIGAR axis as a complementary protective mechanism under oxidative stress.

### 3.3 Overexpression of PKM2 Rescues Dopaminergic Neuron Loss Through Nrf2 Transactivation in an MPTP‐Induced PD Model

**FIGURE 3:**
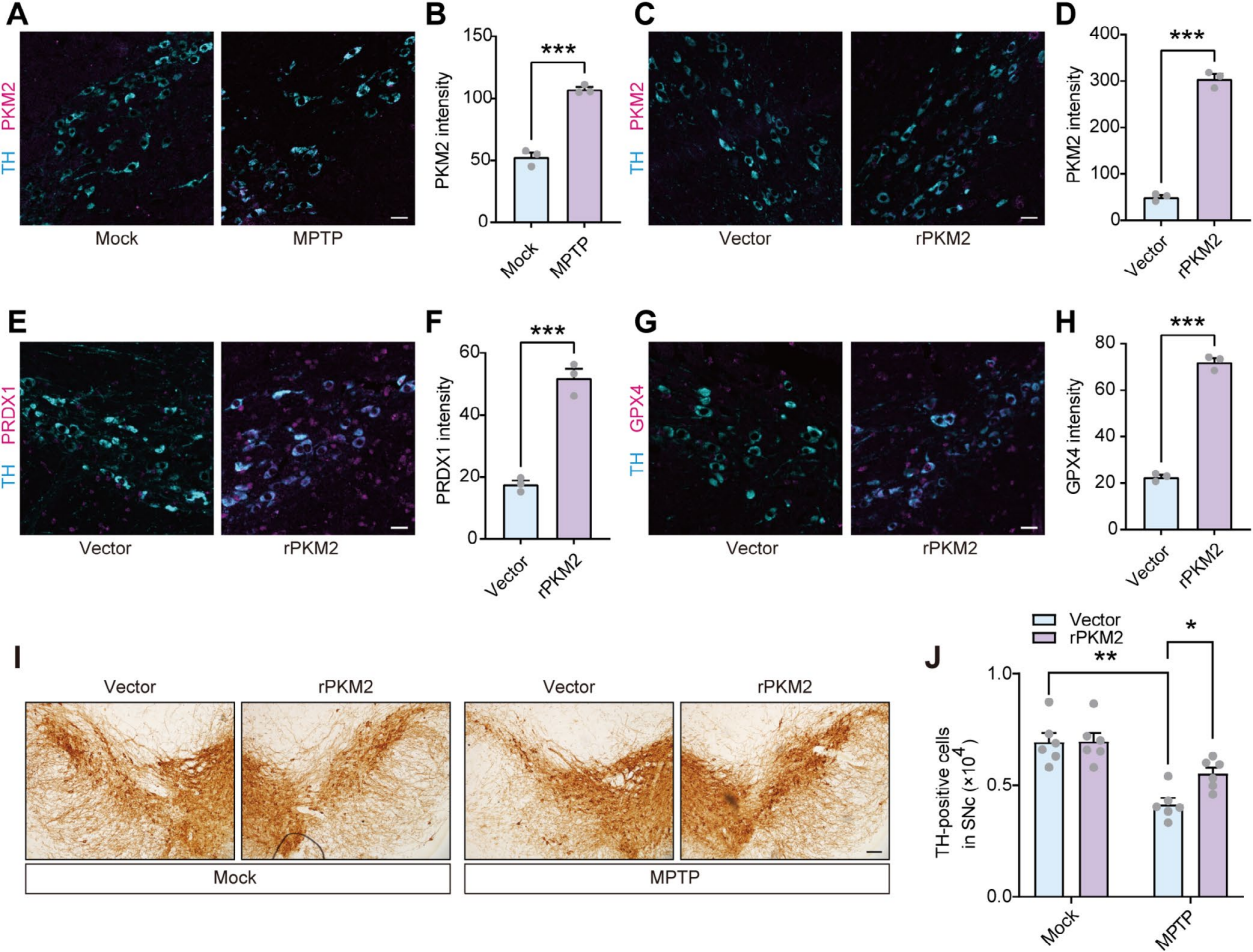
Overexpression of PKM2 rescues dopaminergic neuron loss through Nrf2 transactivation in an MPTP‐induced PD model. (A, B) Representative images and quantification of TSA multispectral staining of PKM2 in TH‐positive neurons from saline or MPTP‐treated mice. *n* = 3 mice per group. Scale bar = 50 μm. (C, D) Representative images and quantification of TSA multispectral staining of PKM2 in TH‐positive neurons from mice that were stereotaxically injected with AAV‐TH‐PKM2 or AAV‐TH‐CTL in the SNc. *n* = 3 mice per group. Scale bar = 50 μm. (E, F) Representative images and quantification of TSA multispectral staining of PRDX1 in TH‐positive neurons from mice that were stereotaxically injected with AAV‐TH‐PKM2 or AAV‐TH‐CTL in the SNc. *n* = 3 mice per group. Scale bar = 50 μm. (G, H) Representative images and quantification of TSA multispectral staining of GPX4 in TH‐positive neurons from mice that were stereotaxically injected with AAV‐TH‐PKM2 or AAV‐TH‐CTL in the SNc. *n* = 3 mice per group. Scale bar = 50 μm. (I, J) Representative images and quantification of immunohistochemistry staining of TH‐positive neurons from saline or MPTP‐treated mice that were stereotaxically injected with AAV‐TH‐PKM2 or AAV‐TH‐CTL in the SNc. *n* = 6 mice per group. Scale bar = 100 μm. *p < 0.05, **p < 0.01, ***p < 0.001. Student's unpaired t test (B, D, F, and H). Two‐way analysis of variance followed by Tukey's post hoc test (J). Data are presented as mean ± SEM. GPX4, Glutathione peroxidase 4; MPTP, 1‐methyl‐4‐phenyl‐1,2,3,6‐tetrahydrodropyridine; PKM2, Pyruvate kinase M2; PRDX1, Peroxiredoxin 1; SNc, Substantia nigra pars compacta; TH, Tyrosine hydroxylase.

We subsequently examined the alterations of PKM2 in the MPTP‐induced PD model. The results revealed a significant increase in PKM2 protein levels in TH‐positive neurons in the MPTP model (Figure 3A,B). Concurrently, we also observed an upregulation of PKM2 expression in primary neurons treated with MPP^+^ (Figure S3A,B). To validate whether the upregulated PKM2 exerts neuroprotective effects through Nrf2 activation, we achieved TH‐positive neuron‐specific overexpression of PKM2 using AAV‐TH‐PKM2 (Figure 3C,D). TSA multispectral staining results demonstrated that overexpressed PKM2 upregulated the expression of Nrf2 target genes, PRDX1 and GPX4 (Figure 3E–H). In primary neurons, overexpressed PKM2 predominantly exists as a dimer (Figure S3I). Consistent with our in vivo findings, PKM2 overexpression also increases the expression of PRDX1 and GPX4 (Figure S3C–H). To more comprehensively evaluate whether PKM2 activates the broader Nrf2‐driven antioxidant program, we performed additional qPCR analyses in primary neurons. The results showed that PKM2 overexpression significantly increased the mRNA levels of multiple canonical Nrf2 target genes, including NQO1, HO‐1, G6PDH, GCLC, GCLM, GPX2, GSR1, and GSTA1, further supporting the role of PKM2 in enhancing Nrf2‐dependent transcriptional activity (Figure S3J). To elucidate whether PKM2 exerts an antioxidant effect by modulating the nuclear translocation of Nrf2, we detected the nuclear and cytoplasmic levels of Nrf2. The immunoblotting results showed that PKM2 overexpression did not alter the abundance of either nuclear or cytoplasmic Nrf2 protein, indicating that PKM2 does not influence Nrf2 nuclear translocation or its overall protein stability (Figure S3K,L). However, ChIP assays demonstrated that PKM2 overexpression significantly enhanced the recruitment of Nrf2 to the promoter regions of PRDX1 and GPX4 (Figure S3M,N). These findings collectively suggest that the upregulated PKM2 in TH‐positive neurons can exert neuroprotection by enhancing the DNA‐binding activity of Nrf2 to transactivate its target genes, rather than regulating its nuclear translocation. Consistent with these molecular changes, we evaluated the impact of PKM2 overexpression on the loss of TH‐positive neurons in the MPTP‐induced PD model. Immunohistochemistry staining revealed that PKM2 overexpression significantly reduced the loss of TH‐positive neurons in the SNc region (Figure 3I,J), a result that mirrored our observations in primary neurons (Figure S3O,P).

To determine whether the PKM2–Nrf2 coupling extends beyond neurons under MPP^+^ stimulation, we examined PKM2 expression and function in glial cells. The immunoblotting results showed that MPP^+^ stimulation did not lead to a significant change of PKM2 within either astrocytes or microglia (Figure S4A–D). To further investigate the role of PKM2 in Nrf2 activation under MPP^+^ stimulation in glial cells, we measured the expression of Nrf2 downstream targets, GPX4 and PRDX1, following PKM2 knockdown and MPP^+^ treatment. qPCR analysis revealed that MPP^+^ treatment led to a higher expression of GPX4 and PRDX1 in both astrocytes and microglia, whereas PKM2 knockdown suppressed this induction (Figure S4E–J). These findings suggest that PKM2‐mediated Nrf2 transactivation is not cell type‐specific and also operates in glial cells, consistent with our previous report of their interaction in astrocytes [39].

### 3.4 MPP ^+^ Stimulation Accelerates PKM Alternative Splicing via Upregulating hnRNP A1/A2

Subsequently, our focus shifted toward elucidating the mechanism underlying the upregulation of PKM2 in TH‐positive neurons in MPTP mice and MPP^+^‐stimulated primary neurons. Given that PKM2 is generated through PKM alternative splicing [51], we initially assessed PKM expression in TH‐positive neurons in MPTP mice and MPP^+^‐treated primary neurons, both in vivo and ex vivo results indicated no significant alterations in PKM protein expression (Figures S5A,B and S6A,B). Notably, neuronal PKM1 exhibited a marked downregulation in the MPTP/MPP^+^ group compared with the control group (Figures S5C,D and S6C,D). In light of the observed upregulation of PKM2 expression, we hypothesized that MPTP/MPP^+^ induces PKM alternative splicing in neurons. To test this hypothesis, we examined PKM gene alternative splicing upon MPP^+^ treatment in primary neurons. Distinguishing PKM1 and PKM2 isoforms through the cleavage of exon 9 with NcoI and exon 10 with PstI, both restriction enzyme digestion and PCR results revealed that MPP^+^ stimulation led to unchanged PKM mRNA expression, upregulated PKM2 mRNA expression, and downregulated PKM1 mRNA expression in primary neurons (Figure S6E,F), thus validating our hypothesis.

**FIGURE 4:**
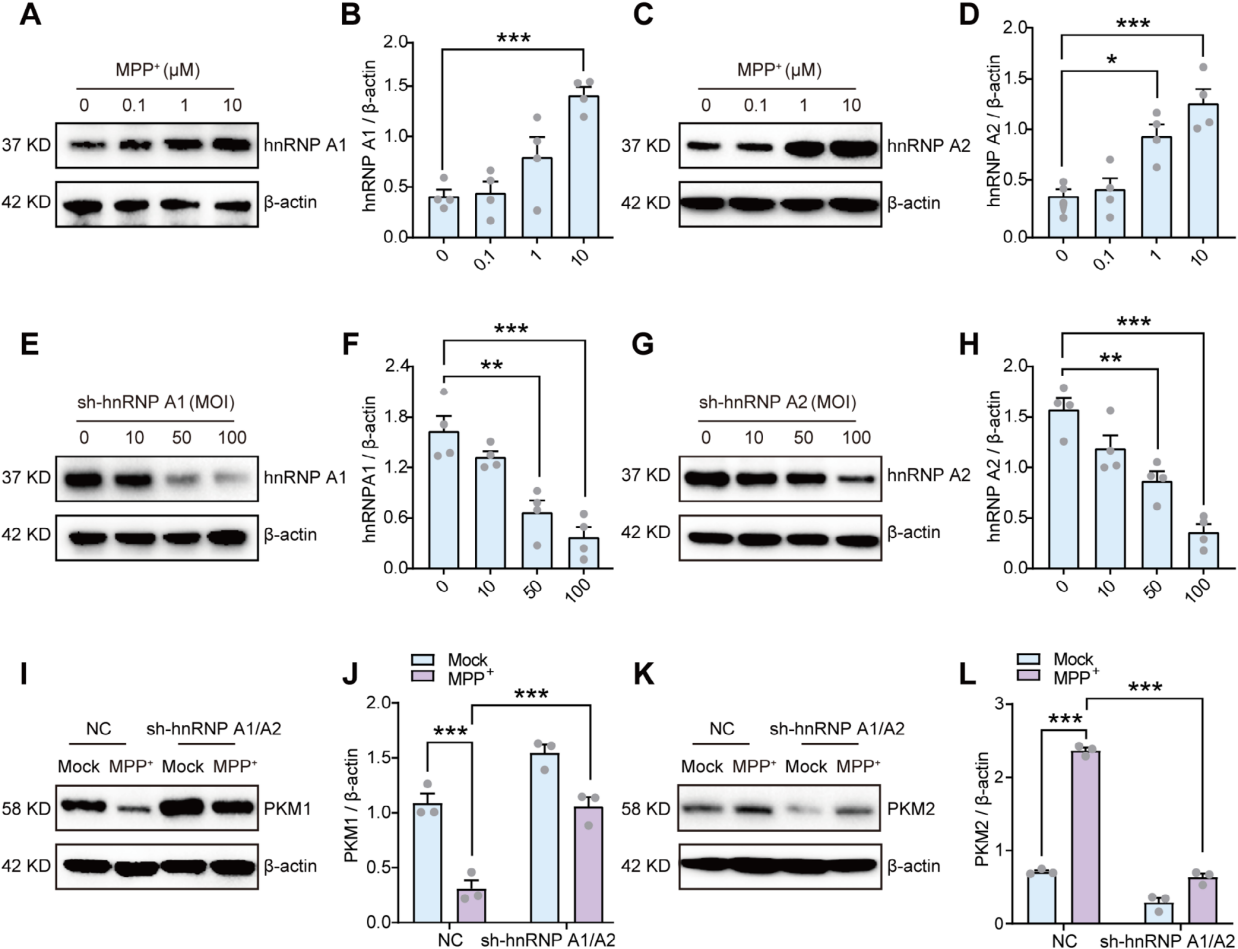
MPP^+^ stimulation accelerates PKM alternative splicing via upregulating hnRNP A1/A2. (A, B) Representative images and quantification of western blot analysis of hnRNP A1 in concentration gradient MPP^+^‐treated primary neurons. (C, D) Representative images and quantification of western blot analysis of hnRNP A2 in concentration gradient MPP^+^‐treated primary neurons. (E, F) Representative images and quantification of western blot analysis of hnRNP A1 in concentration gradient sh‐hnRNP A1‐infected primary neurons. (G, H) Representative images and quantification of western blot analysis of hnRNP A2 in concentration gradient sh‐hnRNP A2‐infected primary neurons. (I, J) Representative images and quantification of western blot analysis of PKM1 in NC or sh‐hnRNP A1/A2‐infected primary neurons treated with or without MPP^+^. (K, L) Representative images and quantification of western blot analysis of PKM2 in NC or sh‐hnRNP A1/A2‐infected primary neurons treated with or without MPP^+^. For three to four independent experiments. *p < 0.05, **p < 0.01, ***p < 0.001. One‐way analysis of variance followed by Tukey's post hoc test (B, D, F, and H). Two‐way analysis of variance followed by Tukey's post hoc test (J and L). Data are presented as mean ± SEM. hnRNP A1, Heterogeneous nuclear ribonucleoprotein A1; hnRNP A2, Heterogeneous nuclear ribonucleoprotein A2; MOI, Multiplicity of infection; MPP^+^, 1‐methyl‐4‐phenylpyridinium; NC, Negative control; PKM1, Pyruvate kinase M1; PKM2, Pyruvate kinase M2.

Previous studies have established that PKM gene splicing is operated by hnRNP A1/A2, which bind to sequences flanking exon 9, ultimately attributed to exon 10 inclusion and PKM2 upregulation [58]. Accordingly, we assessed the expression of hnRNP A1/A2 in MPP^+^‐induced primary neurons and found that MPP^+^ stimulation increased hnRNP A1/A2 expression (Figure 4A–D). This phenomenon was similarly observed in TH‐positive neurons in MPTP mice (Figure S7A–D). Additionally, knocking down hnRNP A1/A2 reduced the extent of MPP^+^‐induced PKM alternative splicing (Figure 4E–L), suggesting that MPP^+^ promotes the transition from PKM1 to PKM2 in neurons by upregulating hnRNP A1/A2.

### 3.5 HnRNP A1/A2 Knockdown Exacerbates Neuronal Death by Suppressing Nrf2 Activation

**FIGURE 5:**
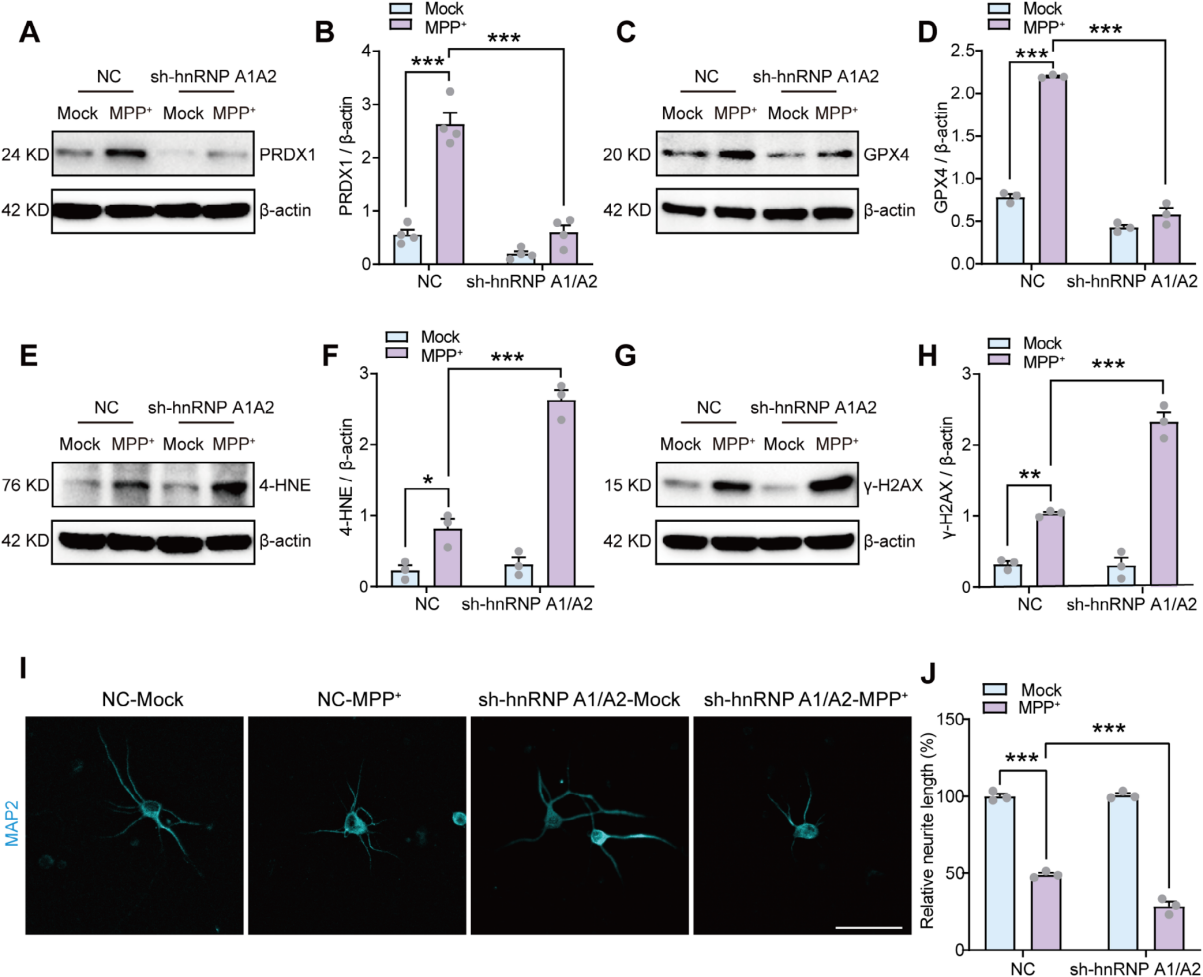
HnRNP A1/A2 knockdown exacerbates neuronal death by suppressing Nrf2 activation. (A, B) Representative images and quantification of western blot analysis of PRDX1 in NC or sh‐hnRNP A1/A2‐infected primary neurons treated with or without MPP^+^. (C, D) Representative images and quantification of western blot analysis of GPX4 in NC or sh‐hnRNP A1/A2‐infected primary neurons treated with or without MPP^+^. (E, F) Representative images and quantification of western blot analysis of 4‐HNE in NC or sh‐hnRNP A1/A2‐infected primary neurons treated with or without MPP^+^. (G, H) Representative images and quantification of western blot analysis of γ‐H2AX in NC or sh‐hnRNP A1/A2‐infected primary neurons treated with or without MPP^+^. (I, J) Representative images and quantification of immunofluorescence staining of MAP2‐positive neurite length in NC or sh‐hnRNP A1/A2‐infected primary neurons treated with or without MPP^+^. Scale bar = 50 μm. For three to four independent experiments. *p < 0.05, **p < 0.01, ***p < 0.001. Two‐way analysis of variance followed by Tukey's post hoc test (B, D, F, H, and J). Data are presented as mean ± SEM. 4‐HNE, 4‐hydroxynonenal; GPX4, Glutathione peroxidase 4; hnRNP A1/A2, Heterogeneous nuclear ribonucleoprotein A1/A2; MPP^+^, 1‐methyl‐4‐phenylpyridinium; NC, Negative control; PRDX1, Peroxiredoxin 1.

Subsequently, we examined the influence of hnRNP A1/A2 knockdown on Nrf2 activation and neuronal injury induced by MPP^+^. Immunoblotting revealed that hnRNP A1/A2 knockdown hindered the elevated protein expression of Nrf2 targets PRDX1 and GPX4 triggered by MPP^+^ treatment (Figure 5A–D), consequently accelerating the generation of the lipid peroxidation product, 4‐HNE (Figure 5E,F). Furthermore, hnRNP A1/A2 knockdown exacerbated MPP^+^‐induced primary neuron death, as evidenced by increased γ‐H2AX accumulation (Figure 5G,H) and a further reduction in neurite length (Figure 5I,J). Collectively, these findings suggest that hnRNP A1/A2 knockdown exacerbates MPP^+^‐induced oxidative stress and neuronal loss by disrupting the PKM2‐mediated Nrf2 activation pathway.

### 3.6 MPP ^+^ Induces Upregulation of hnRNP A1/A2 Expression via ROS Induction

**FIGURE 6:**
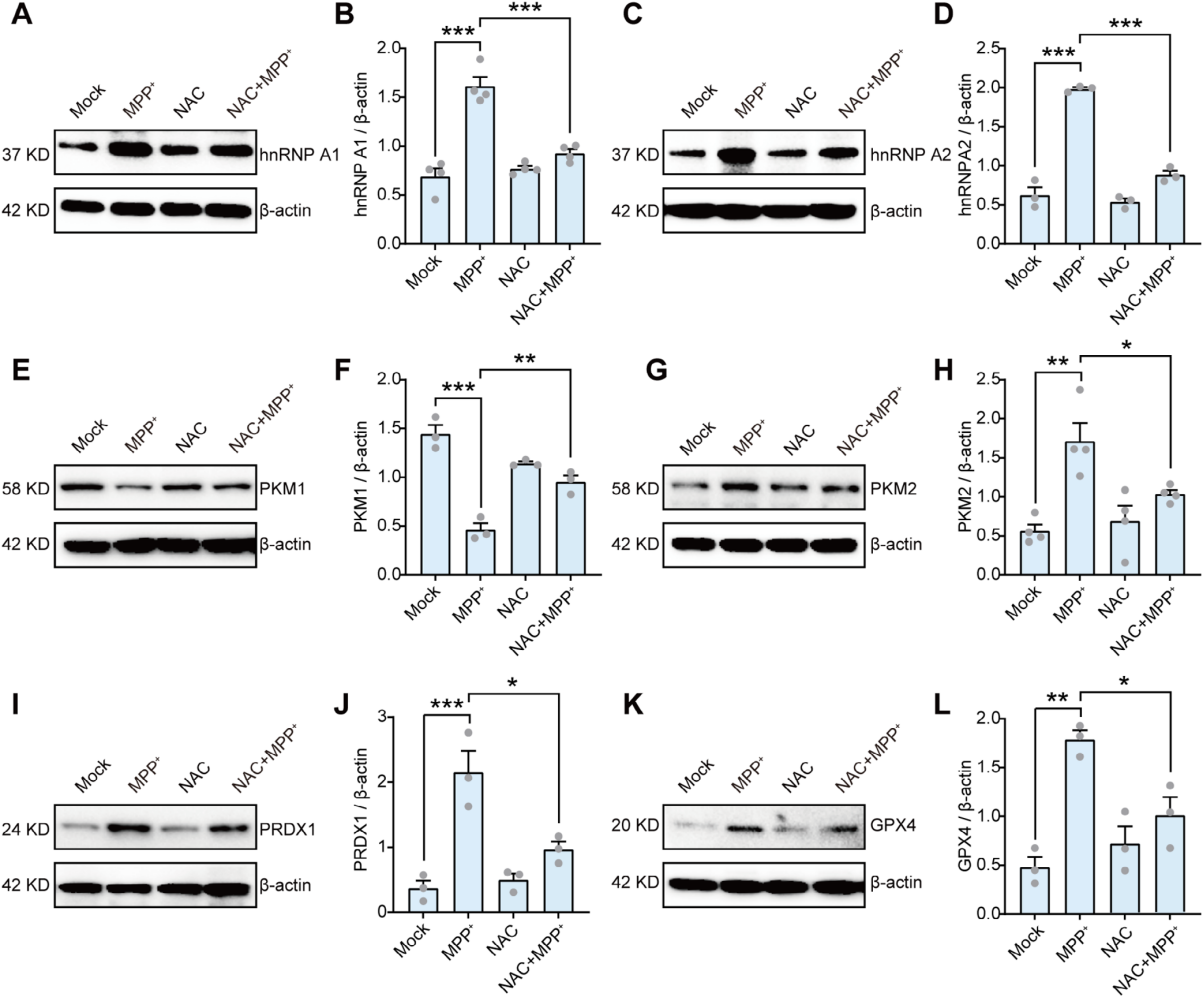
MPP^+^ induces upregulation of hnRNP A1/A2 expression via reactive oxygen species induction. (A, B) Representative images and quantification of western blot analysis of hnRNP A1 in PBS or NAC preconditioned primary neurons treated with or without MPP^+^. (C, D) Representative images and quantification of western blot analysis of hnRNP A2 in PBS or NAC preconditioned primary neurons treated with or without MPP^+^. (E, F) Representative images and quantification of western blot analysis of PKM1 in PBS or NAC preconditioned primary neurons treated with or without MPP^+^. (G, H) Representative images and quantification of western blot analysis of PKM2 in PBS or NAC preconditioned primary neurons treated with or without MPP^+^. (I, J) Representative images and quantification of western blot analysis of PRDX1 in PBS or NAC preconditioned primary neurons treated with or without MPP^+^. (K, L) Representative images and quantification of western blot analysis of GPX4 in PBS or NAC preconditioned primary neurons treated with or without MPP^+^. For three to four independent experiments. *p < 0.05, **p < 0.01, ***p < 0.001. One‐way analysis of variance followed by Tukey's post hoc test (B, D, F, H, J, and L). Data are presented as mean ± SEM. GPX4, Glutathione peroxidase 4; hnRNP A1, Heterogeneous nuclear ribonucleoprotein A1; hnRNP A2, Heterogeneous nuclear ribonucleoprotein A2; MPP^+^, 1‐methyl‐4‐phenylpyridinium; NAC, N‐Acetylcysteine; PKM1, Pyruvate kinase M1; PKM2, Pyruvate kinase M2; PRDX1, Peroxiredoxin 1.

As MPP^+^ inhibits complex I of the mitochondrial respiratory chain and generates ROS, we hypothesized that the ROS produced by MPP^+^ contributes to the increased expression of hnRNP A1/A2. To test this hypothesis, we pre‐treated primary neurons with NAC, an ROS scavenger, followed by MPP^+^ exposure. Immunoblotting results revealed a significant reduction in hnRNP A1 and hnRNP A2 protein levels in the NAC combined with MPP^+^ group compared with the MPP^+^ group (Figure 6A–D). Consequently, the expression of PKM1 and PKM2 was also affected. NAC administration led to the upregulation of PKM1 and downregulation of PKM2 compared with the MPP^+^ group by eliminating the ROS generated by MPP^+^ (Figure 6E–H). Subsequent examination of the antioxidants PRDX1 and GPX4 showed that NAC preconditioning suppressed the MPP^+^‐induced increase in the expression of these two proteins (Figure 6I–L). Collectively, these findings confirm that MPP^+^‐induced ROS production increases hnRNP A1/A2 expression, thereby promoting PKM alternative splicing in primary neurons.

## 4 Discussion

PD is a neurodegenerative disease with complex pathogenesis, which is mainly characterized by a significant reduction in dopamine levels in the brain. Current therapeutic drugs directly or indirectly focus on increasing dopamine levels in the brain. Given their propensity for side effects and questionable long‐term benefits, researchers have proposed therapeutic strategies beyond dopamine [59]. In our previous studies, we have found that PKM2 in astrocytes activates Nrf2, which promotes the synthesis of the antioxidant molecule glutathione [39]. In the current study, we also observed that under MPTP stimulation, neuronal PKM2 is upregulated and activates Nrf2. By exploring the effects of overexpression and knockdown of neuronal PKM2 in PD, we discovered a protective role of neuronal PKM2, suggesting that both astrocytic and neuronal PKM2 play regulatory roles in the onset and development of PD. This effect of PKM2 on the progression of PD is mediated by the activation of Nrf2 against oxidative damage, which constitutes a therapeutic approach beyond dopamine, supporting the proposal that PKM2 represents a potential therapeutic target for antioxidant intervention in PD. However, further preclinical and clinical studies are required to verify the precise function of PKM2 in PD.

Importantly, our neuron‐focused analyses add several layers of context beyond prior astrocyte findings. Dopaminergic neurons are the primary pathological substrate in PD, and we provide neuron‐intrinsic in vivo evidence linking PKM2 to oxidative injury and dopaminergic neuron loss in the MPTP model. Moreover, neurons and astrocytes differ markedly in metabolic demand and redox buffering capacity [60, 61, 62], such that the regulation and functional consequences of PKM2 signaling in neurons cannot be assumed from glial studies. Finally, our data reveal a stress‐inducible endogenous compensatory mechanism in neurons in which ROS upregulates hnRNP A1/A2, drives PKM alternative splicing toward PKM2, and thereby reinforces Nrf2‐dependent antioxidant transcription.

PKM2 exists in two configurations, dimeric and tetrameric, with its conformational changes regulated by allosteric modulators including synthetic small molecules [63], natural products [64], and intermediates of glycolysis [65, 66, 67], as well as post‐translational modifications such as O‐GlcNAcylation [68], phosphorylation [69], and S‐nitrosylation [70]. The different configurations of PKM2 exhibit distinct functions, with the tetrameric form displaying higher pyruvate kinase activity compared to the dimeric form. In non‐neuronal cells, like tumor cells, endothelial cells, or during acute kidney injury, the antioxidative function of PKM2 is primarily achieved through conformational changes. Oxidation or S‐nitrosylation modifications cause PKM2 to transform from a tetramer to a dimer, thereby inhibiting its kinase activity and reprogramming the glycolytic metabolic flow to the pentose phosphate pathway to support antioxidant defense [70, 71, 72]. In neurons, our findings revealed that overexpressed PKM2 predominantly exists as a dimeric configuration, which aligns with its non‐canonical function as a transcriptional coactivator. This dimeric PKM2 activates Nrf2 to counteract oxidative damage. Mechanistically, the antioxidative role of PKM2 in neurons involves a complete signaling cascade encompassing both upstream regulation and downstream effector functions. Upstream, MPTP/MPP^+^‐induced ROS upregulate the expression of alternative splicing factors hnRNP A1 and hnRNP A2, which promote PKM alternative splicing toward PKM2 and enhance its cellular abundance. Downstream, dimeric PKM2 exerts its neuroprotective effects through direct physical interaction with Nrf2 and by enhancing the binding of Nrf2 to antioxidant response elements in the promoters of target genes. These genes collectively reinforce antioxidant defense and glutathione (GSH) biosynthetic pathways, directly scavenging ROS, and ultimately protecting neurons from oxidative stress‐induced degeneration. Additionally, this PKM2‐mediated Nrf2 transactivation occurs independently of Nrf2 nuclear translocation, as evidenced by unaltered nuclear Nrf2 abundance following PKM2 overexpression. This further highlights the unique role of dimeric PKM2 as a transcriptional coactivator in neurons.

Beyond its non‐canonical role as a coactivator, PKM2's classical glycolytic function is pivotal in neurological diseases [37]. In the present study, under PD pathological conditions, ROS drive PKM gene alternative splicing, converting high‐activity PKM1 to low‐activity PKM2. This shift redirects glucose metabolites into the pentose phosphate pathway, which generates NADPH. Concurrently, Nrf2 target genes GCLC and GCLM, rate‐limiting enzymes for GSH synthesis, depend on PPP‐derived NADPH to maintain GSH in its reduced, active antioxidant form [39, 73, 74]. This metabolic‐antioxidant crosstalk indicates the dual role of PKM2 in reshaping neuronal metabolism and reinforcing redox defense, positioning it as a central coordinator of adaptive responses to oxidative stress in PD.

Furthermore, the PKM2–Nrf2 pathway may intersect with core PD‐related pathogenic cascades involving α‐synuclein accumulation and elevated LRRK2 kinase activity—two well‐recognized drivers that converge to trigger excessive reactive oxygen species production. This reactive oxygen species further exacerbates α‐synuclein misfolding and aggregation while amplifying LRRK2 hyperactivation, forming a self‐reinforcing vicious cycle that accelerates dopaminergic neurodegeneration [75, 76, 77, 78]. By scavenging excess reactive oxygen species, the PKM2–Nrf2‐mediated antioxidant response holds the potential to interrupt this pathogenic loop, thereby mitigating α‐synuclein aggregation and LRRK2‐related neurotoxicity. Future studies in genetic PD models such as A53T transgenic mice and LRRK2 G2019S knock‐in mice will directly validate this hypothesis, reinforcing PKM2's potential as a disease‐modifying target that integrates with well‐established PD pathogenic pathways.

HnRNP A1 and hnRNP A2 are members of the hnRNP family, primarily localized in the cell nucleus, where they participate in various metabolic processes including RNA splicing, transport, transcription, and translation regulation by recognizing and binding to specific RNA substrates [79, 80]. They are involved in the occurrence and development of neurodegenerative diseases such as amyotrophic lateral sclerosis, frontotemporal lobar degeneration, multiple sclerosis, spinal muscular atrophy, Alzheimer's disease, and Huntington's disease [80, 81, 82, 83]. Neuronal hnRNP A1/A2 play roles in regulating gene expression and maintaining neuronal morphology and function [84, 85]. Studies have shown that abnormal aggregation of hnRNP A1/A2, caused by mutations in their prion‐like domains, is a significant contributor to the progressive loss of motor neurons [86]. Apart from abnormal aggregation, nucleocytoplasmic mislocalization of hnRNP A1, leading to decreased nuclear and increased cytoplasmic expression of hnRNP A1, is an important molecular mechanism underlying neuronal death and the progress of diseases such as amyotrophic lateral sclerosis and multiple sclerosis [87, 88, 89]. Notably, weakened cholinergic signaling results in the downregulation of hnRNP A1/A2 in Alzheimer's disease, disrupting the splicing of proteins crucial for cortical neuronal function and ultimately leading to synapse loss and impairment of learning and memory functions [90]. These findings underscore the significant roles of hnRNP A1 and hnRNP A2 in neurons. Our study indicates that the upregulation of hnRNP A1/A2 expression in dopaminergic neurons promotes the alternative splicing of the PKM gene, thereby upregulating PKM2 expression, which protects dopaminergic neurons from oxidative damage.

## 5 Conclusions

In summary, we found that the dopamine neurons exhibited significant upregulation of PKM2 in the MPTP‐induced mouse model of PD. The mechanism underlying this upregulation phenomenon is orchestrated by the heightened level of the PKM alternative splicing regulators, hnRNP A1 and hnRNP A2, induced by MPTP/MPP^+^‐generated ROS, which promote PKM alternative splicing, resulting in the downregulation of PKM1 and the upregulation of PKM2. The upregulation of PKM2 in dopamine neurons transcriptionally activates Nrf2, enhancing the expression of its target genes PRDX1 and GPX4, thereby counteracting oxidative damage and exerting neuroprotective effects in PD (Figure S8).

## Author Contributions

Meng Mei performed the in vitro experiments and drafted the manuscript. Qian‐qian Miao conducted the in vivo experiments and drafted the manuscript. Hao‐ran Wang and Meng‐ke Li assisted in experimental procedures and data curation. Jian‐hua Ding supervised the study. Gang Hu and Yao Wei conceived and supervised the study, and edited the manuscript.

## Funding

This work was supported by National Natural Science Foundation of China (82003738, 81991523), The Funding for Scientific Research Projects from Wuhan Municipal Health Commission (WX23A43).

## Ethics Statement

All animal experimental protocols were reviewed and approved by the Institutional Animal Care and Use Committee of Huazhong University of Science and Technology (Approval ID: IACUC Number: 3404).

## Conflicts of Interest

The authors declare no conflicts of interest.

## Supporting information


**Figure S1:** Deletion efficiency of PKM2 in TH‐positive neurons in the SNc.
**Figure S2:** Effect of PKM2 silencing on the expression of SOD2 and TIGAR in MPP^+^‐treated SH‐SY5Y cells.
**Figure S3:** Upregulated PKM2 activates Nrf2 to protect neurons from MPP^+^‐induced damage.
**Figure S4:** Effect of MPP^+^ on glial cells in terms of PKM2 expression and Nrf2 activation.
**Figure S5:** Expression of PKM and PKM1 in an MPTP‐induced PD model.
**Figure S6:** MPP^+^ induces PKM alternative splicing in primary neurons.
**Figure S7:** Expression of hnRNP A1 and hnRNP A2 is increased in an MPTP‐induced PD model.
**Figure S8:** Schematic diagram illustrating the role of PKM2 in dopaminergic neurons.

## Data Availability

The data that support the findings of this study are available from the corresponding author upon reasonable request.
