# Supplementary material for: Upregulated PKM2 Protects Dopaminergic Neurons From Oxidative Damage Through Nrf2 Transactivation in an MPTP‐Induced Mouse Model of Parkinson's Disease
Source: CNS Neurosci Ther. 2026 Jun 1;32(6):e70968. doi: 10.1002/cns.70968 (PMC13239746; doi:10.1002/cns.70968)
Supplement: Supplementary file 1 — Figure S1: Deletion efficiency of PKM2 in TH‐positive neurons in the SNc. Figure S2: Effect of PKM2 silencing on the expression of SOD2 and TIGAR in MPP+‐treated SH‐SY5Y cells. Figure S3: Upregulated PKM2 activates Nrf2 to protect neurons from MPP+‐induced damage. Figure S4: Effect of MPP+ on glial cells in terms of PKM2 expression and Nrf2 activation. Figure S5: Expression of PKM and PKM1 in an MPTP‐induced PD model. Figure S6: MPP+ induces PKM alternative splicing in primary neurons. Figure S7: Expression of hnRNP A1 and hnRNP A2 is increased in an MPTP‐induced PD model. Figure S8: Schematic diagram illustrating the role of PKM2 in dopaminergic neurons. [file CNS-32-e70968-s001.docx]

**SUPPLEMENTARY MATERIALS**

**
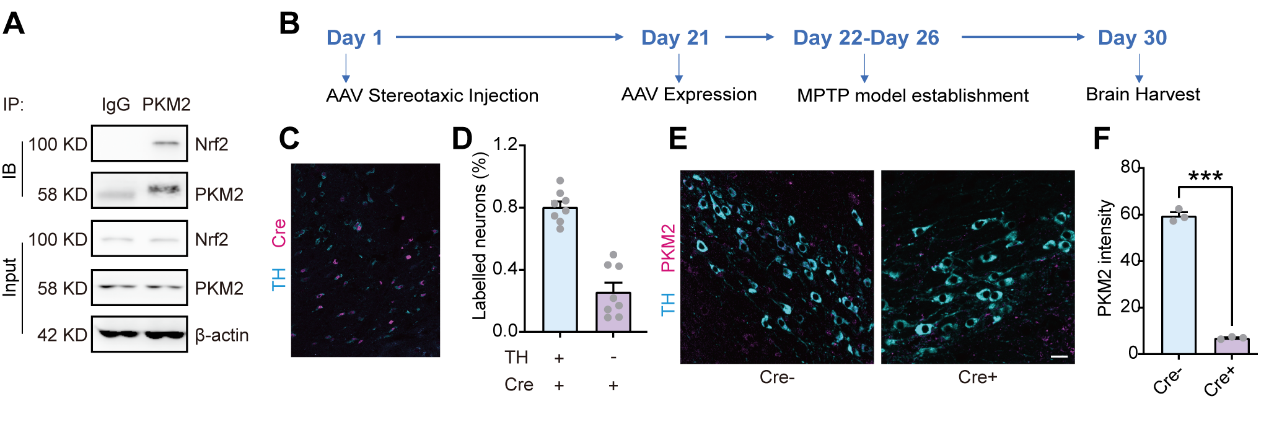
**

**FIGURE S1 Deletion efficiency of PKM2 in TH-positive neurons in the SNc**

**(A)** Representative images of Co-IP assay of PKM2-Nrf2 interaction in primary neurons. **(B)** Schematic timeline of the animal experimental procedure. **(C, D)** Representative images and quantification of TSA multispectral staining of Cre in TH-positive neurons in PKM2^flox/flox^ mice that were stereotaxically injected with AAV-TH-Cre in the SNc. n=8 mice per group. (**E, F)** Representative images and quantification of TSA multispectral staining of PKM2 in TH-positive neurons in PKM2^flox/flox^ mice that were stereotaxically injected with AAV-TH-Cre or AAV-TH-CTL in the SNc. n=3 mice per group. Scale bar = 50 μm. ***p<0.001. Student’s unpaired t test (F). Data are presented as mean ± SEM. PKM2: pyruvate kinase M2; Nrf2: nuclear factor erythroid 2-related factor 2, TH: tyrosine hydroxylase.


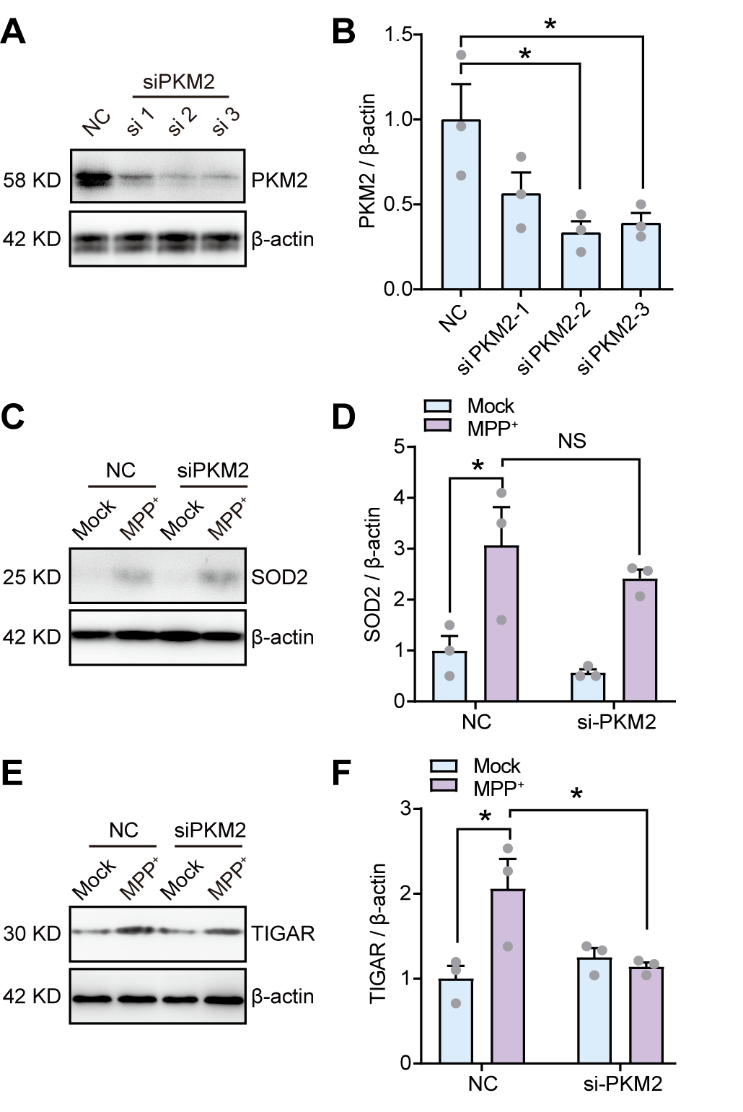


**FIGURE S2 Effect of PKM2 silencing on the expression of SOD2 and TIGAR in MPP⁺-treated SH-SY5Y cells**

**(A, B)** Representative images and quantification of western blot analysis of PKM2 in SH-SY5Y transfected with NC or PKM2 siRNAs. **(C, D)** Representative images and quantification of western blot analysis of SOD2 in NC or si PKM2-transfected SH-SY5Y treated with or without MPP^+^. **(E, F)** Representative images and quantification of western blot analysis of TIGAR in NC or si PKM2-transfected SH-SY5Y treated with or without MPP^+^. For three independent experiments. *p<0.05. One-way analysis of variance followed by Tukey’s post-hoc test (B). Two-way analysis of variance followed by Tukey’s post-hoc test (D and F). Data are presented as mean ± SEM. MPP^+^: 1-methyl-4-phenylpyridinium; PKM2: pyruvate kinase M2; NC: negative control; NS: not significant; SOD2: Superoxide dismutase 2; TIGAR: TP53-induced glycolysis and apoptosis regulator.


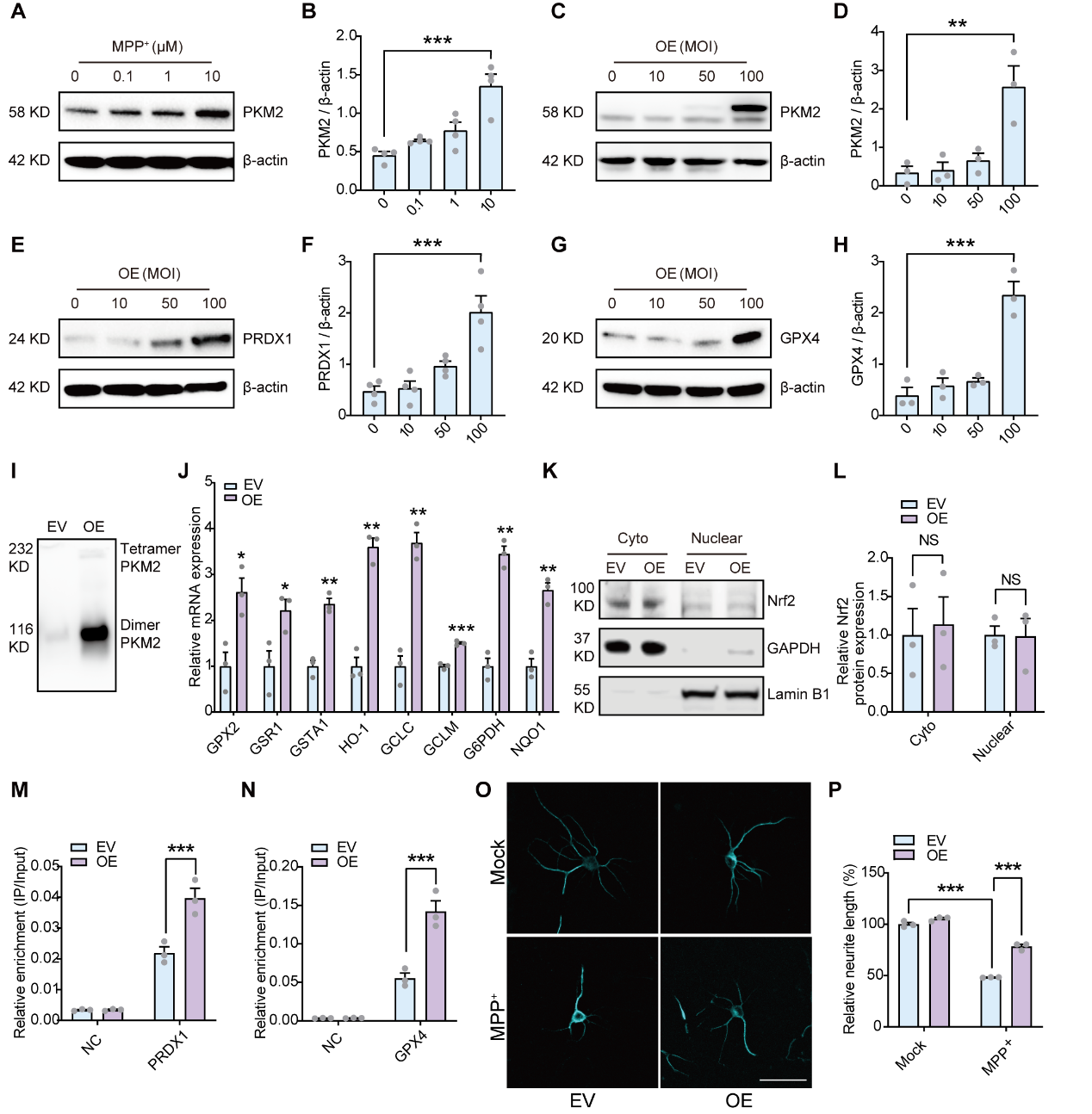


**FIGURE S3 Upregulated PKM2 activates Nrf2 to protect neurons from MPP^+^-induced damage**

**(A, B)** Representative images and quantification of western blot analysis of PKM2 in concentration gradient MPP^+^-treated primary neurons. (**C, D)** Representative images and quantification of western blot analysis of PKM2 in concentration gradient AV-PKM2-OE-infected primary neurons. (**E, F)** Representative images and quantification of western blot analysis of PRDX1 in concentration gradient AV-PKM2-OE-infected primary neurons. (**G, H)** Representative images and quantification of western blot analysis of GPX4 in concentration gradient AV-PKM2-OE-infected primary neurons. **(I)** Representative images of dimeric and tetrameric PKM2 in AV-EV or AV-PKM2-OE-infected primary neurons. **(J)** PCR quantification of Nrf2 targets mRNA levels in AV-EV or AV-PKM2-OE-infected primary neurons. **(K, L)** Representative images and quantification of western blot analysis of cytoplasmic and nuclear Nrf2 in AV-EV or AV-PKM2-OE-infected primary neurons. **(M, N)** ChIP assays showing Nrf2 enrichment at PRDX1 (M) and GPX4 (N) promoters in AV-EV or AV-PKM2-OE-infected primary neurons. **(O, P)** Representative images and quantification of immunofluorescence staining of MAP2-positive neurite length in AV-EV or AV-PKM2-OE-infected primary neurons treated with or without MPP^+^. Scale bar = 50 μm. For three to four independent experiments. *p<0.05, **p<0.01, ***p<0.001. One-way analysis of variance followed by Tukey’s post-hoc test (B, D, F, and H). Two-way analysis of variance followed by Tukey’s post-hoc test (P). Student’s unpaired t test (J, L, M and N). Data are presented as mean ± SEM. PKM2: pyruvate kinase M2; OE: overexpression; MOI: multiplicity of infection; PRDX1: peroxiredoxin 1; GPX4: glutathione peroxidase 4; MPP^+^: 1-methyl-4-phenylpyridinium; MAP2: microtubule-associated protein 2; EV: empty vector; NS: not significant; Nrf2: Nuclear factor erythroid 2-related factor 2.


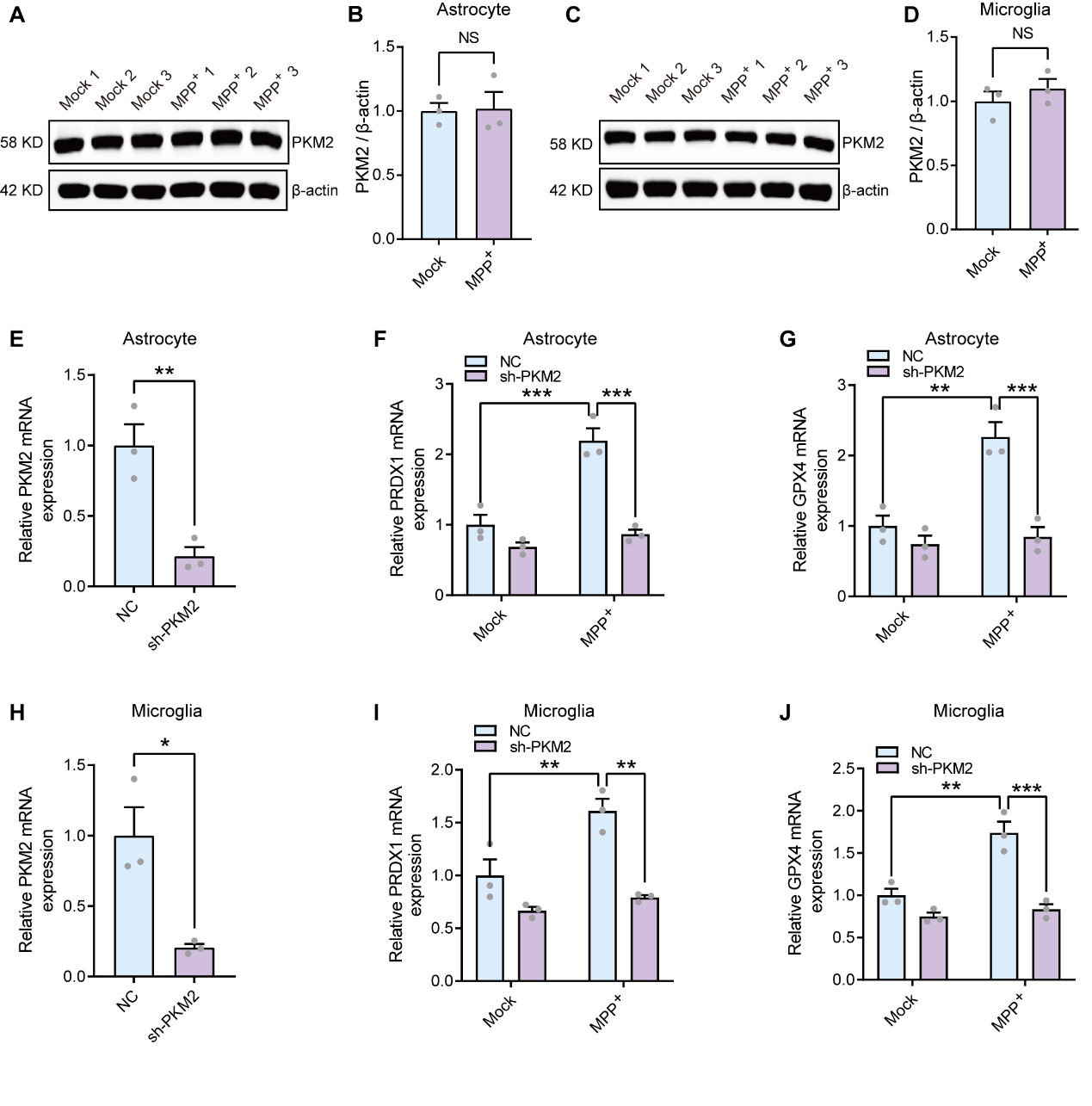


**FIGURE S4 Effect of MPP^+^ on glial cells in terms of PKM2 expression and Nrf2 activation**

**(A, B)** Representative images and quantification of western blot analysis of PKM2 in MPP^+^-treated primary astrocytes. **(C, D)** Representative images and quantification of western blot analysis of PKM2 in MPP^+^-treated primary microglia. **(E)** PCR quantification of PKM2 mRNA levels in NC or sh-PKM2-transfected primary astrocytes. **(F)** PCR quantification of PRDX1 mRNA levels in NC or sh-PKM2-transfected primary astrocytes treated with or without MPP^+^. **(G)** PCR quantification of GPX4 mRNA levels in NC or sh-PKM2-transfected primary astrocytes treated with or without MPP^+^. **(H)** PCR quantification of PKM2 mRNA levels in NC or sh-PKM2-treated primary microglia. **(I)** PCR quantification of PRDX1 mRNA levels in NC or sh-PKM2-transfected primary microglia treated with or without MPP^+^. **(J)** PCR quantification of GPX4 mRNA levels in NC or sh-PKM2-transfected primary microglia treated with or without MPP^+^. For three independent experiments. *p<0.05, **p<0.01, ***p<0.001. Student’s unpaired t test (B, D, E, and H). Two-way analysis of variance followed by Tukey’s post-hoc test (F, G, I, and J). Data are presented as mean ± SEM. MPP^+^: 1-methyl-4-phenylpyridinium; NS: not significant; PKM2: pyruvate kinase M2; NC: negative control; PRDX1: peroxiredoxin 1; GPX4: glutathione peroxidase 4.

**
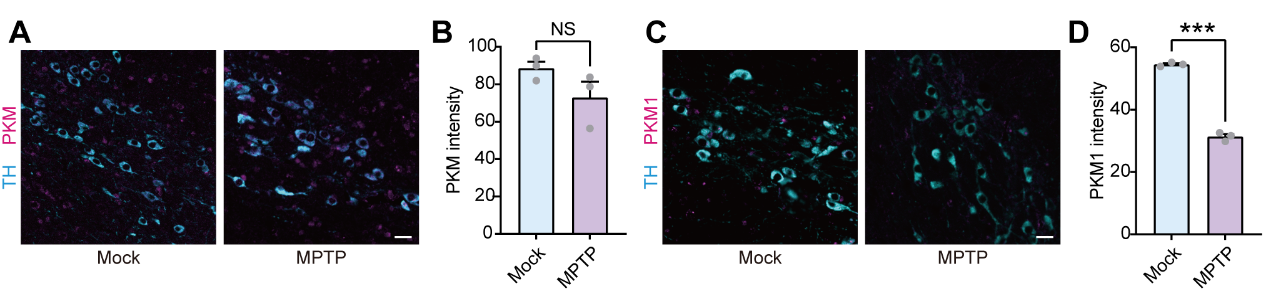
**

**FIGURE S5 Expression of PKM and PKM1 in an MPTP-induced PD model**

**(A, B)** Representative images and quantification of TSA multispectral staining of PKM in TH-positive neurons from saline or MPTP-treated mice. **(C, D)** Representative images and quantification of TSA multispectral staining of PKM1 in TH-positive neurons from saline or MPTP-treated mice. n=3 mice per group. Scale bar = 50 μm. ***p<0.001. Student’s unpaired t test (B and D). Data are presented as mean ± SEM. NS: not significant; TH: tyrosine hydroxylase; MPTP: 1-methyl-4-phenyl-1,2,3,6-tetrahydrodropyridine; PKM: pyruvate kinase M; PKM1: pyruvate kinase M1.

**
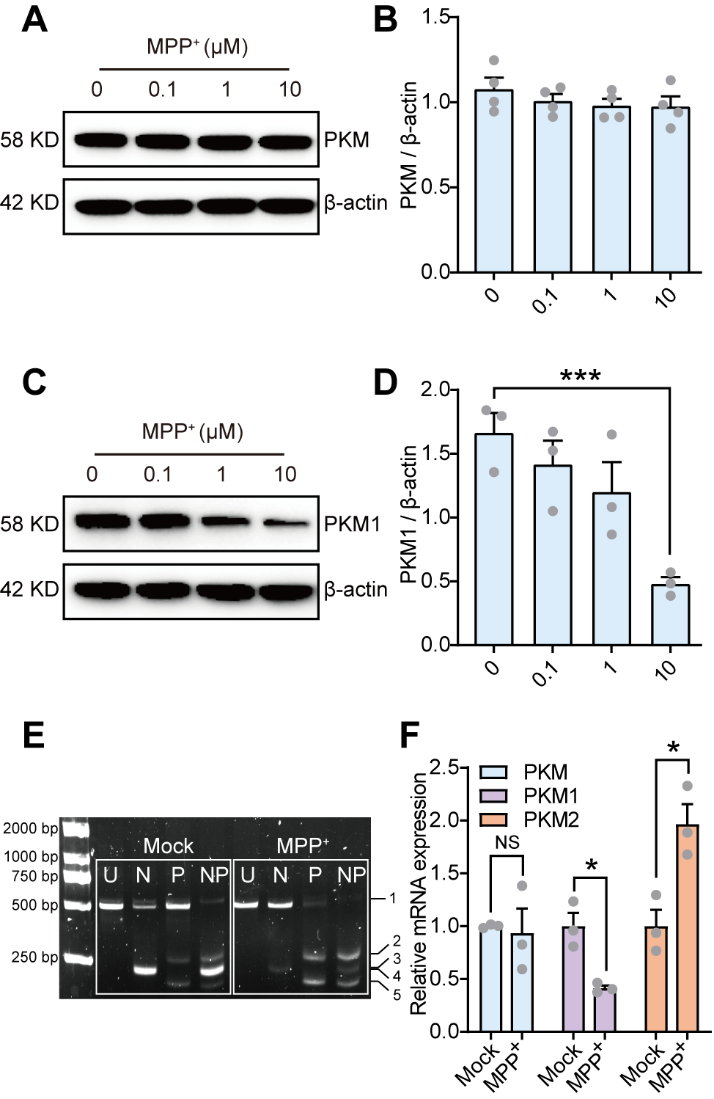
**

**FIGURE S6 MPP^+^ induces PKM alternative splicing in primary neurons**

**(A, B)** Representative images and quantification of western blot analysis of PKM in concentration gradient MPP^+^-treated primary neurons. **(C, D)** Representative images and quantification of western blot analysis of PKM1 in concentration gradient MPP^+^-treated primary neurons. (**E)** Total RNA of PBS or MPP^+^-treated primary neurons were analyzed by RT-PCR followed by digestion with PstI (P), NcoI (N), none (U), or both N and P (NP). The numbered bands are as follows: 1: uncut PKM1 or PKM2 (502 bp); 2: P-cleaved PKM2 5’ fragment (286 bp); 3: N-cleaved PKM1 5’ fragment (245 bp); 4: N-cleaved PKM1 3’ fragment (240 bp); and 5: P-cleaved PKM2 3’ fragment (216 bp). (**F)** PCR quantification of PKM/PKM1/PKM2 mRNA levels in PBS or MPP^+^-treated primary neurons. For three to four independent experiments. *p<0.05, ***p<0.001. One-way analysis of variance followed by Tukey’s post-hoc test (B and D). Student’s unpaired t test (F). Data are presented as mean ± SEM. MPP^+^: 1-methyl-4-phenylpyridinium; PKM: pyruvate kinase M; PKM1: pyruvate kinase M1; PKM2: pyruvate kinase M2; NS: not significant.

**
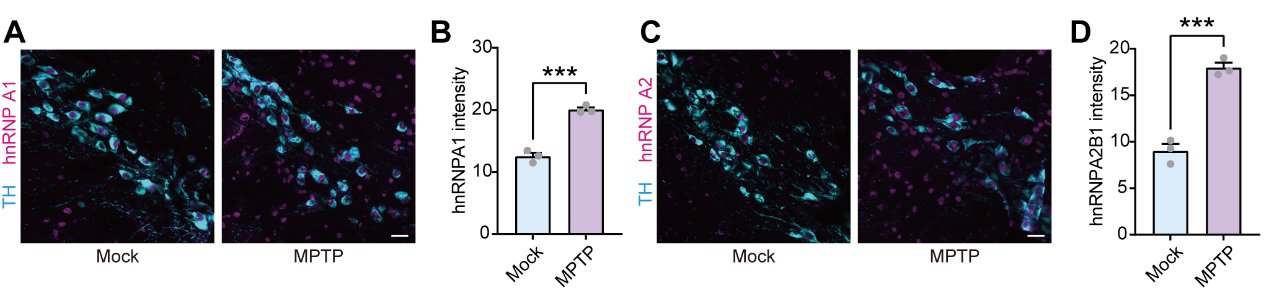
**

**FIGURE S7 Expression of hnRNP A1 and hnRNP A2 is increased in an MPTP-induced PD model**

**(A, B)** Representative images and quantification of TSA multispectral staining of hnRNP A1 in TH-positive neurons from saline or MPTP-treated mice. **(C, D)** Representative images and quantification of TSA multispectral staining of hnRNP A2 in TH-positive neurons from saline or MPTP-treated mice. n=3 mice per group. Scale bar = 50 μm. ***p<0.001. Student’s unpaired t test (B and D). Data are presented as mean ± SEM. TH: tyrosine hydroxylase; MPTP: 1-methyl-4-phenyl-1,2,3,6-tetrahydrodropyridine; hnRNP A1: heterogeneous nuclear ribonucleoprotein A1; hnRNP A2: heterogeneous nuclear ribonucleoprotein A2.

**
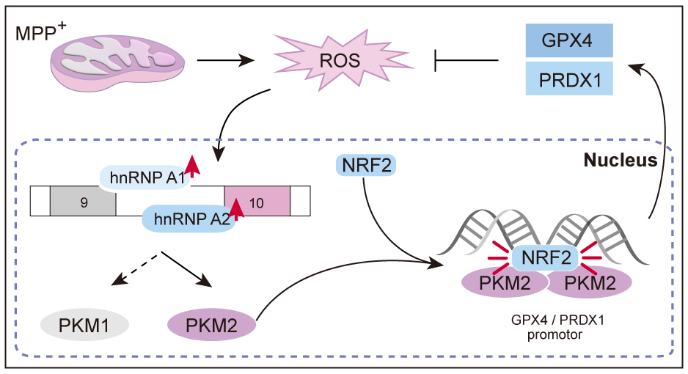
**

**FIGURE S8 Schematic diagram illustrating the role of PKM2 in dopaminergic neurons**

MPP^+^-produced reactive oxygen species upregulate hnRNP A1/A2 to promote PKM alternative splicing, which in turn increases PKM2 expression and activates Nrf2. Upregulated PKM2 protected dopaminergic neurons from oxidative damage through Nrf2 transactivation in the MPTP-induced PD model.
